# Tuberculosis treatment intermittency in the continuation phase and mortality in HIV-positive persons receiving antiretroviral therapy

**DOI:** 10.1186/s12879-022-07330-5

**Published:** 2022-04-05

**Authors:** Brenda Crabtree-Ramirez, Cathy A. Jenkins, Bryan E. Shepherd, Karu Jayathilake, Valdilea G. Veloso, Gabriela Carriquiry, Eduardo Gotuzzo, Claudia P. Cortes, Dennis Padgett, Catherine McGowan, Juan Sierra-Madero, Serena Koenig, Jean W. Pape, Timothy R. Sterling, the CCASAnet Region of IeDEA, Pedro Cahn, Carina Cesar, Valeria Fink, Zulma Ortiz, Florencia Cahn, Agustina Roldan, Ines Aristegui, Claudia Frola, Beatriz Grinsztejn, Valdilea G. Veloso, Paula M. Luz, Sandra Cardoso Wagner, Ruth Friedman, Ronaldo I. Moreira, Lara Esteves Coelho, Monica Derrico Pedrosa, Guilherme Amaral Calvet, Hugo Perazzo, Rodrigo Moreira, Maria Pia Diniz Ribeiro, Mario Sergio Pereira, Emilia Moreira Jalil, Jorge Pinto, Flavia Ferreira, Marcelle Maia, Aida de Fátima Barbosa Gouvêa, Fabiana do Carmo, Claudia Cortes, Marcelo Wolff, Maria Fernanda Rodriguez, Gabriel Castillo, Gladys Allendes, Jean William Pape, Vanessa Rouzier Adias Marcelin, Youry Macius, Stephano Saint Preux, Serena Koenig, Marco Tulio Luque Diana Varela, Magda Chavez, Ada Mailhot, Denis Padgett, Juan Sierra Madero, Brenda Crabtree Ramirez, Yanink Caro Vega, Eduardo Gotuzzo, Fernando Mejia, Gabriela Carriquiry, Catherine CMcGowan, Stephany N. Duda, Bryan E. Shepherd, Timothy Sterling, Anna K Person, Peter F. Rebeiro, Jessica Castilho, William C. Wester, Kate Clouse, Karu Jayathilake, Fernanda Maruri Hilary Vansell, Marina Cruvinel Figueiredo, Cathy Jenkins, Ahra Kim Sarah Lotspeich, Paridhi Ranadive, Kate Clouse

**Affiliations:** 1 Departamento de Infectología. Instituto Nacional de Ciencias Médicas Y Nutrición Salvador Zubirán, Mexico City, Mexico; 2 Vanderbilt University Medical Center, A2209 Medical Center North, 1161 21st Avenue South, Nashville, TN 37232 USA; 3 Instituto Nacional de Infectologia Evandro Chagas, Fundacao Oswaldo Cruz, Rio de Janeiro, Brasil; 4 Instituto de Medicina Tropical Alexander Von Humboldt, Universidad Peruana Cayetano Heredia, Lima, Peru; 5 Fundación Arriarán, University of Chile, Santiago, Chile; 6 Hospital Escuela and Instituto Hondureño de Seguridad Social, Tegucigalpa, Honduras; 7 Division of Global Health Equity, Brigham and Women’s Hospital, Boston, MA USA; 8 Le Groupe Haïtien d’Etude du Sarcome de Kaposi Et Des Infections Opportunistes (GHESKIO), Port-au-Prince, Haiti

**Keywords:** Tuberculosis, Tuberculosis treatment, HIV, Intermittent treatment, ART, TB maintenance treatment

## Abstract

**Background:**

Some tuberculosis (TB) treatment guidelines recommend daily TB treatment in both the intensive and continuation phases of treatment in HIV-positive persons to decrease the risk of relapse and acquired drug resistance. However, guidelines vary across countries, and treatment is given 7, 5, 3, or 2 days/week. The effect of TB treatment intermittency in the continuation phase on mortality in HIV-positive persons on antiretroviral therapy (ART), is not well-described.

**Methods:**

We conducted an observational cohort study among HIV-positive adults treated for TB between 2000 and 2018 and after enrollment into the Caribbean, Central, and South America network for HIV epidemiology (CCASAnet; Brazil, Chile, Haiti, Honduras, Mexico and Peru). All received standard TB therapy (2-month initiation phase of daily isoniazid, rifampin or rifabutin, pyrazinamide ± ethambutol) and continuation phase of isoniazid and rifampin or rifabutin, administered concomitantly with ART. Known timing of ART and TB treatment were also inclusion criteria. Kaplan–Meier and Cox proportional hazards methods compared time to death between groups. Missing model covariates were imputed via multiple imputation.

**Results:**

2303 patients met inclusion criteria: 2003(87%) received TB treatment 5–7 days/week and 300(13%) 2–3 days/week in the continuation phase. Intermittency varied by site: 100% of patients from Brazil and Haiti received continuation phase treatment 5–7 days/week, followed by Honduras (91%), Peru (42%), Mexico (7%), and Chile (0%). The crude risk of death was lower among those receiving treatment 5–7 vs. 2–3 days/week (HR = 0.68; 95% CI = 0.51—0.91; P = 0.008). After adjusting for age, sex, CD4, ART use at TB diagnosis, site of TB disease (pulmonary vs. extrapulmonary), and year of TB diagnosis, mortality risk was lower, but not significantly, among those treated 5–7 days/week vs. 2–3 days/week (HR 0.75, 95%CI 0.55–1.01; P = 0.06). After also stratifying by study site, there was no longer a protective effect (HR 1.42, 95%CI 0.83–2.45; P = 0.20).

**Conclusions:**

TB treatment 5–7 days/week was associated with a marginally decreased risk of death compared to TB treatment 2–3 days/week in the continuation phase in multivariable, unstratified analyses. However, little variation in TB treatment intermittency within country meant the results could have been driven by other differences between study sites. Therefore, randomized trials are needed, especially in heterogenous regions such as Latin America.

**Supplementary Information:**

The online version contains supplementary material available at 10.1186/s12879-022-07330-5.

## Background

Intermittent therapy has been widely used in the continuation phase of first-line tuberculosis (TB) treatment, reducing medication and healthcare worker costs for TB programs, and facilitating global scale-up of directly observed therapy. [1–4] However, given the emergence of TB drug resistance and concerns regarding TB relapse, the World Health Organization (WHO) has recommended daily therapy as the preferred intermittency for TB treatment since 2008 [5, 6]. In addition, the American Thoracic Society (ATS), Centers for Disease Control and Prevention (CDC), and Infectious Diseases Society of America (IDSA) recommended daily TB treatment for HIV-positive persons in 2016 [7]. However, guidelines vary across regions and countries, and treatment may be given 7, 5, 3, or 2 days/week in the continuation phase. For example, in Mexico and Argentina, TB treatment guidelines for HIV-positive persons recommend daily therapy during the initial two-month intensive phase and thrice-weekly therapy in the continuation phase [8–10]. In Brazil and Haiti, national recommendations include daily therapy in the continuation phase [11–14]. In Peru, previous national guidelines recommended intermittent treatment in the continuation phase, but more recently, daily therapy has been recommended [14].

Few studies have described the effect of TB treatment intermittency in the continuation phase on mortality in HIV-positive persons, particularly in persons receiving concomitant antiretroviral therapy (ART). In TB patients without HIV, systematic reviews have evaluated clinical outcomes according to different dosing frequencies. [15–17] Johnston and collaborators published a meta-analysis demonstrating that thrice-weekly dosing throughout TB treatment was associated with increased mortality compared to daily therapy. [16] Less frequent dosing schedules were significantly associated with an increased risk of TB relapse. In the meta-regression, they were also significantly associated with higher relapse rates, failure, and acquired drug resistance [16].

Several randomized, controlled trials (RCTs) have been published addressing first-line standard TB treatment, with few focusing on the intermittency of continuation phase treatment in HIV-negative patients [18–21]. More recently, a randomized clinical trial performed at several sites in South India concluded that in HIV-positive patients, daily anti-TB therapy throughout treatment for pulmonary TB was superior to thrice-weekly treatment throughout therapy, and thrice-weekly treatment in the continuation phase (with daily therapy in the intensive phase) [22]. However, the sample size was small, and hepatoxicity risk was higher with daily therapy. Additionally, a prospective observational study concluded that thrice-weekly anti-TB therapy was effective in HIV- negative but not HIV-positive patients [23]. Patients who received directly observed intermittent anti-TB therapy during the intensive phase had a 40% higher risk of mortality than patients who received an unsupervised daily regimen [24]. Our group published an observational study assessing factors associated with mortality in TB/HIV co-infected patients. Duration of more than 6 months of TB treatment was associated with better survival than 6 months of treatment [25]. However, the description of frequency of intermittency in the continuation phase of TB treatment in HIV-positive persons in Latin America and its impact in survival has not been evaluated; data from large cohorts could provide important information for all settings, to confirm the data from the one clinical trial above. Given current recommendations that TB and HIV should be treated concomitantly, we focused on persons who received TB treatment and ART.

## Methods

### Cohort and overview

This study was performed in the Caribbean, Central and South America network for HIV Epidemiology (CCASAnet; http://ccasanet.vanderbilt.edu), which has been described elsewhere [26]. The collaboration was established in 2006 as part of IeDEA (International epidemiology Databases to Evaluate AIDS, https://www.iedea.org/) to collect data from HIV-positive persons in care in Central and South America and the Caribbean. CCASAnet sites from Brazil, Chile, Haiti, Honduras, Mexico, and Peru were included. All sites are large, centralized, urban and public clinics; TB treatment was provided free of charge.

Institutional review board approval was obtained locally for each participating site and the CCASAnet data coordinating center (DCC) at Vanderbilt University Medical Center, Nashville, TN, USA. At each of the sites contributing data to this study, except IMTAvH-Peru, ethical regulations and policies permit retrospective analysis of de-identified clinical data without informed consent when research is approved by an Institutional Review Board or appropriately constituted ethics committee. At IMTAvH-Peru, patients consent at the time of enrollment to provide de-identified clinical data for research studies.

### Study design and population

All HIV-positive adults (≥ 18 years) diagnosed with TB on or after enrollment in CCASAnet and between 2000 and 2018 and whose initiation phase treatment for TB included: isoniazid (INH), rifampin (RIF) or rifabutin (RBT), and pyrazinamide (PZA), with or without ethambutol (EMB), were included in the analysis. Patients were followed until the first of last recorded date on which they were known to be alive prior to December 31, 2019 or their date of death. Tuberculosis treatment in the continuation phase had INH and RIF or RBT. Patients were categorized according to the frequency of TB treatment intermittency in the continuation phase (2–3/week vs. 5–7/week), following a recent meta-analysis [16]. Concomitant ART was determined by comparing the date of TB diagnosis and the end of the continuation phase of treatment with the ART start and stop dates; if there was any overlap, patients were considered to have received concomitant TB treatment and ART and included in the analysis. We excluded TB diagnoses prior to CCASAnet enrollment and patients with missing data on treatment intermittency, or whose ART was not concomitant with TB treatment. Only the first episode of TB after enrollment was included in the analysis. The date of TB diagnosis was the date of TB treatment initiation.

Missing treatment initiation phase stop dates were singly imputed to be 60 days after the treatment initiation start date; date of death or last day of follow-up if < 60 days. Missing treatment continuation phase stop dates were singly imputed to be 180 days after the treatment continuation phase start date; date of death or last day of follow-up if < 180 days.

### Outcome and statistical analysis

Descriptive statistics on the entire cohort as well as those who had non-missing intermittency data were reported as median (interquartile range [IQR]) and percent (frequency), as appropriate. The primary outcome was time to death since TB diagnosis.

Unadjusted Kaplan–Meier survival curves and multivariable Cox proportional hazards models were used to investigate the association between time from TB diagnosis to death and frequency of continuation phase treatment (5–7x/week (referent) vs. 2–3x/week). Cox models included both unstratified and stratified by study site (i.e., the stratified Cox model allowed the baseline hazard to vary by site, but still estimated common regression coefficients pooled across sites) [25]. Cox models were adjusted for CD4 (square root transformed) and age at TB diagnosis, sex, site of TB (pulmonary only versus any extrapulmonary), timing of ART relative to the TB diagnosis (not on ART at TB diagnosis versus on ART at TB diagnosis), and year of TB diagnosis. CD4 at TB diagnosis was defined as the closest CD4 to the date of TB diagnosis within a window of 180 days before to 30 days after the diagnosis. Age, CD4 at TB diagnosis, and year of TB diagnosis were fit with restricted cubic splines (4 knots) to relax linearity assumptions with the specific point estimates informed by the distribution of our data. Missing model covariates were imputed using predictive mean matching (5 imputation replications). Univariate and multivariable models (complete case and multiply imputed analyses) were done. Sensitivity analyses of the Kaplan Meier curves were also done among those with pulmonary TB only, and among those receiving treatment in Peru. Statistical analyses were performed using R Statistical Software, Version 3.5.2 (www.R-project.org). Analysis code is posted at https://biostat.app.vumc.org/ArchivedAnalyses.

All methods were carried out in accordance with relevant guidelines and regulations.

## Results

### Population studied

**Fig. 1:**
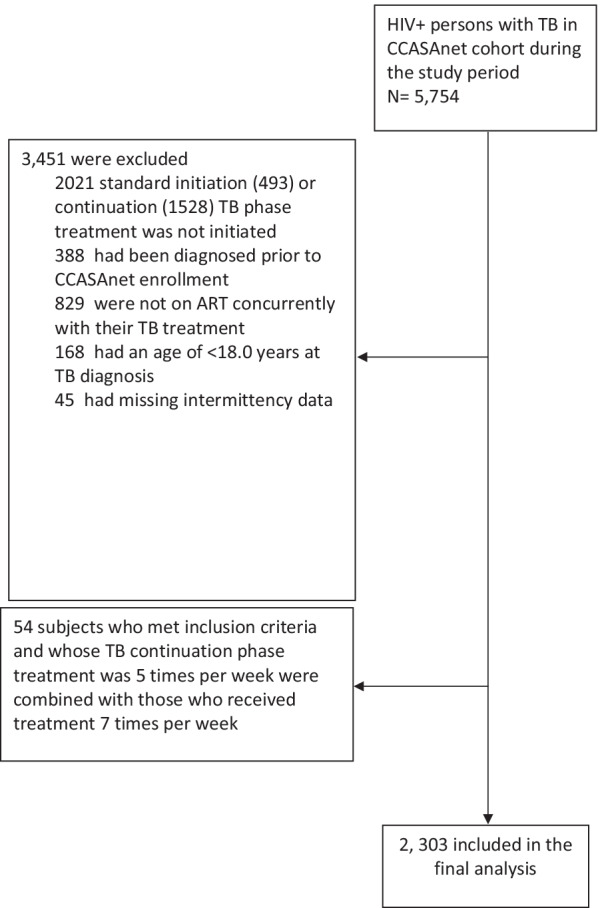
Reasons for exclusion from the study population

There were 5754 HIV-positive TB patients in the CCASAnet cohort during the study period, of whom 3733 started standard 3- or 4-drug initial phase TB therapy and received isoniazid plus a rifamycin in the continuation phase. Of these, 2303 had a TB diagnosis after enrollment, were ≥ 18 years of age at that diagnosis, received concurrent TB treatment and ART, and had continuation phase treatment data available (Fig. 1). Clinical and demographic characteristics of those excluded vs. included in the analysis are shown in the supplemental material (Additional file 1). The median follow-up time was 4.0 (IQR 1.30, 7. 10) years. There were 300 (13%) patients who received TB treatment 2–3 days per week and 2003 (87%) who received TB treatment 5–7 days per week in the continuation phase. (54 and 1949 patients received TB treatment 5 and 7 days per week, respectively) Table 1 provides the clinical and demographic characteristics of the overall study population, and according to treatment intermittency. Intermittency varied by site: 100% of patients from Brazil and Haiti received continuation phase treatment 5–7 days/week, followed by Honduras (91%), Peru (42%), Mexico (7%), and Chile (0%).

**Table 1:** Clinical and demographic characteristics of the study population according to TB treatment intermittency frequency in the continuation phase

	**N**	**2-3x/wk**	**5-7x/wk**	**Combined**	**P-value**
		**N = 300**	**N = 2003**	**N = 2303**	
Age at TB diagnosis—median (IQR)	2303	34.80 (28.95, 42.61)	36.61 (29.97, 44.82)	36.41 (29.72, 44.38)	0.021
Male sex	2303	84% (251)	55% (1107)	59% (1358)	< 0.001
CD4 at TB diagnosis—median (IQR)	1602	97 (43, 196)	173 (67, 310)	156.5 (60.0, 295)	< 0.001
Missing baseline CD4 indicator	2303				< 0.001
Not missing		91% (273)	66% (1329)	70% (1602)	
Missing		9% (27)	34% (674)	30% (701)	
Baseline HIV-1 RNA (log10-transformed)	438	5.15 (4.70, 5.64)	5.10 (4.55, 5.60)	5.11 (4.63, 5.62)	0.609
Missing baseline HIV-1 RNA indicator	2303				< 0.001
Not missing		58% (175)	13% (263)	19% (438)	
Missing		42% (125)	87% (1740)	81% (1865)	
Site of TB	2303				< 0.001
Pulmonary only		54% (163)	78% (1556)	75% (1719)	
Any extrapulmonary		45% (135)	22% (443)	25% (578)	
Unknown		1% (2)	0% (4)	0% (6)	
CCASAnet site	2303				< 0.001
Brazil		0% (0)	12% (245)	11% (245)	
Chile		12% (35)	0% (0)	2% (35)	
Haiti		0% (0)	78% (1560)	68% (1560)	
Honduras		1% (2)	1% (21)	1% (23)	
Mexico		8% (25)	0% (2)	1% (27)	
Peru		79% (238)	9% (175)	18% (413)	
TB relative to ART	2303				0.047
Not on ART at TB diagnosis		65% (196)	59% (1188)	60% (1384)	
On ART at TB diagnosis		35% (104)	41% (815)	40% (919)	
Days on ART at TB diagnosis	919	131.5 (28.5, 401.5)	108 (3, 566.5)	113 (8, 543)	0.272
Days to ART after TB diagnosis	1384	46 (26.00, 79.25)	28 (14, 63)	30 (14, 67)	< 0.001
Days on TB treatment	2303	245 (180, 298)	194 (182, 224)	195 (182, 236)	< 0.001
Days from ART initiation post-TB dx to TB continuation stop	1384	184 (130.8, 251)	168 (131, 190)	168 (131, 197)	< 0.001
Missing Initiation phase stop date	2303				< 0.001
Non-missing		92% (275)	100% (1999)	99% (2274)	
Missing		8% (25)	0% (4)	1% (29)	
Missing Continuation phase stop date	2303				< 0.001
Non-missing		87% (260)	97% (1946)	96% (2206)	
Missing		13% (40)	3% (57)	4% (97)	
Died	2303				< 0.001
No		79% (238)	89% (1783)	88% (2021)	
Yes		21% (62)	11% (220)	12% (282)	
Follow-up time (yrs)	2303	6.55 (3.79, 8.89)	3.61 (1.17, 6.49)	4.03 (1.33, 7.13)	< 0.001

*IQR* inter-quartile range, *ART* antiretroviral therapy, Wilcoxon Rank Sum test used for continuous covariates; Pearson chi-square test used for categorical variables

There were 2303 persons who met inclusion criteria

### Mortality and associated factors

**Fig. 2:**
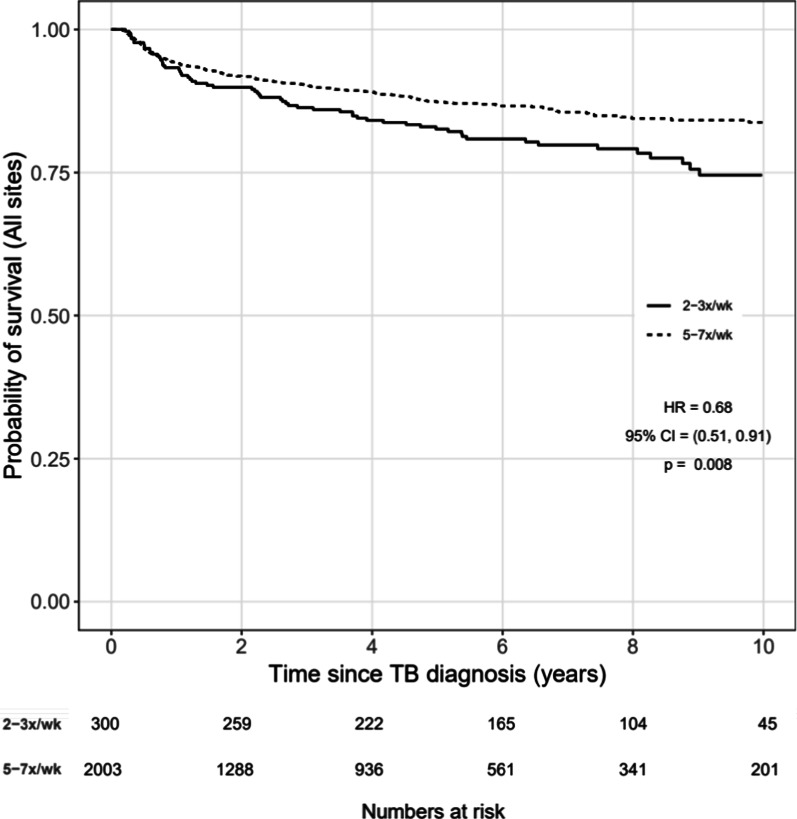
Survival curves dichotomized by continuation phase treatment frequency

Among the 2303 TB patients included in the study, there were 282 deaths: (62 in the 2–3 days/week group and 220 in the 5–7 days/week group). Figure 2 shows the survival curves according to the frequency of TB treatment in the continuation phase; the crude risk of death was lower among the 5–7/week group (HR = 0.68; 95% CI = 0.51—0.91; P = 0.008). After adjusting for age and CD4 at TB diagnosis, sex, site of TB (pulmonary only or any extrapulmonary), timing of ART, and year of TB diagnosis, the multivariable analysis that was not stratified by study site continued to demonstrate a reduction in mortality risk among persons who received TB treatment 5–7 days/week in the continuation phase (HR = 0.75, 95% confidence interval [CI] 0.55–1.01, p = 0.06) (Table 2). However, in the multivariable analysis stratified by study site, the hazard of death was elevated in the 5–7 days/week group (HR = 1.42, 95%CI 0.83–2.45; p = 0.2), though it was not statistically significant (Table 2).

**Table 2:** Survival Analysis. Multivariable Cox Proportional Hazards Models

Covariate	Not stratified by study site			Stratified by study site		
	HR	95% CI	P value	HR	95% CI	P value
Continuation phase frequency						
2–3/week (ref)	1.00	–	0.02	1.00		0.97
5–7/week	0.71	0.52, 0.96		1.01	0.62, 1.63	
Age at TB diagnosis (years)			0.02			0.01
30	1.08	0.97, 1.19		1.07	0.97, 1.19	
35 (ref)	1.00			1.00		
40	1.07	0.90, 1.26		1.07	0.90, 1.27	
45	1.21	0.92, 1.57		1.22	0.93, 1.59	
50	1.37	1.03, 1.82		1.41	1.06, 1.87	
Male sex	0.90	0.70, 1.16	0.41	0.89	0.68, 1.15	0.36
CD4 at TB diagnosis			< 0.001			< 0.001
100	1.25	1.13, 1.39		1.25	1.13, 1.39	
150 (ref)	1.00			1.00		
200	0.82	0.73, 0.92		0.82	0.73, 0.92	
350	0.57	0.42, 0.77		0.56	0.42, 0.76	
Site of TB						
Pulmonary only	1.00	–	0.48	1.00	–	0.77
Extrapulmonary TB	0.91	0.69, 1.19		0.96	0.70, 1.30	
ART relative to TB diagnosis						
Not on ART	1.00	–	0.05	1.00	–	0.14
On ART	1.28	1.00, 1.63		1.21	0.94, 1.56	

Analyses were performed both without and with stratification by study site; the latter to account for other potential differences by study site (n = 2288). Missing data were imputed via multiple imputation

*ART* antiretroviral therapy

In the stratified and unstratified multivariable analysis, increasing CD4 was associated with a decreased risk of death (p < 0.001 in both models). In contrast, increasing age was associated with an increased risk of death (p ≤ 0.01 in both models). In addition, being on ART at TB diagnosis was associated with an increased hazard of death when not stratifying by site (HR = 1.30, 95% CI 1.02–1.66, p = 0.03); however, this association was not statistically significant in the stratified analysis (HR = 1.23, 95% CI 0.96–1.59, p = 0.11). The risk of death decreased with more recent year of TB diagnosis (Table 2).

### Pulmonary tuberculosis

**Fig. 3:**
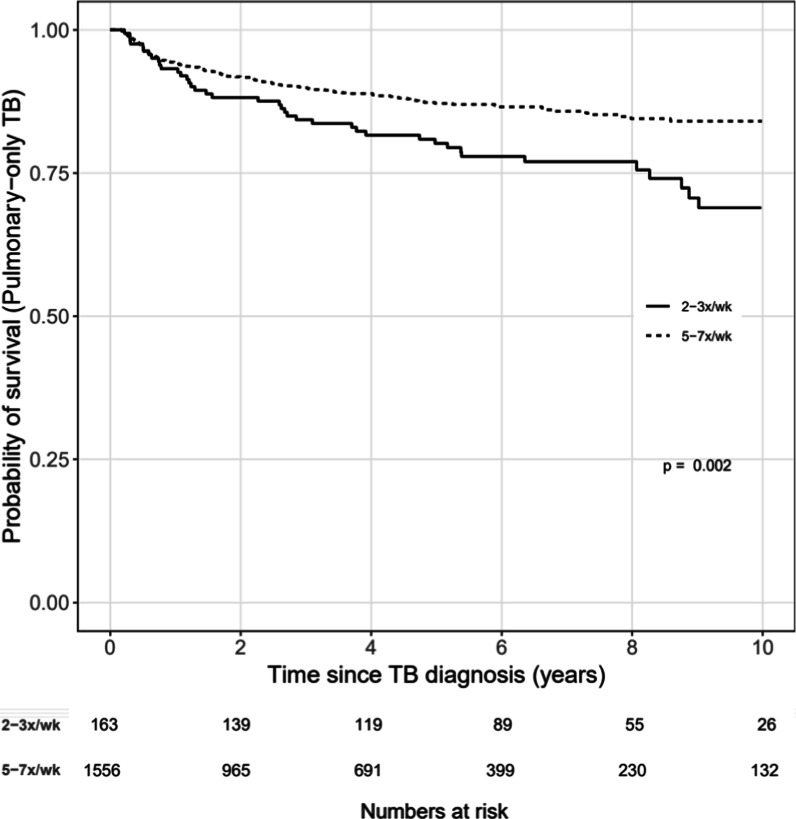
Survival curves dichotomized by contination pase treatment frequency

There were 1719 (75%) patients with pulmonary TB only; 91% received continuation phase treatment 5–7 times per week, and they were mainly from Haiti, Brazil, and Peru. The Kaplan Meier curve among the pulmonary-only cohort showed that those with continuation phase treatment 5–7 times per week had higher survival than those in the 2–3 times per week group (p = 0.002) (Fig. 3).

### Analysis limited to Peru

**Fig. 4:**
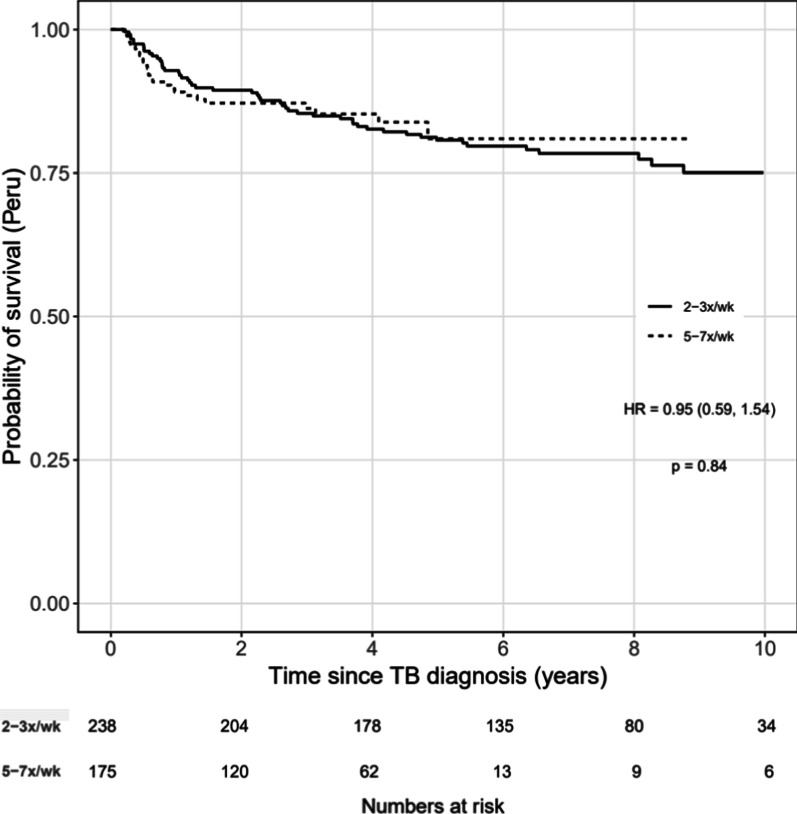
Survival curves by dichotomized continuation pase frequency at the study site in Peru

The study site with the greatest diversity of treatment intermittency in the continuation phase was Peru. There were 175 persons who received treatment 5–7 times per week and 238 who received treatment 2–3 times per week. In a Kaplan–Meier analysis of these participants, the survival between the two groups was similar (uHR = 0.95, 95% CI = 0.59–1.54, p = 0.84; Fig. 4).

## Discussion

In the present study, we assessed for differences in mortality in HIV-positive patients with TB depending on the intermittency of TB treatment in the continuation phase. There was a decreased mortality risk in patients who received treatment 5–7 days/week compared to 2–3 days/week, as evidenced by the crude risk of death in the Kaplan–Meier curves, and the multivariable Cox model that adjusted for age, sex, CD4 count, ART use at TB diagnosis, site of TB disease, and year of TB diagnosis. However, after stratifying by study site, there was no longer a protective effect. This suggests that there may have been other differences among sites that accounted for the difference in survival; this could include factors such as differences in ART, host factors, or access to specialized care. As expected, increased age and decreased CD4 count at diagnosis were statistically significant risk factors for mortality across all analyses. The risk of death increased even after TB treatment completion, which we have noted previously [26].

There was substantial variation across our sites in access to ART, treatment practices, and resources for treating HIV and TB co-infection. For example, sites had varying access to ART drug classes such as integrase strand transfer inhibitors, including raltegravir and more recently, dolutegravir. In addition, differences in HIV drug resistance patterns and resultant ART regimens could have also affected adherence and clinical outcomes [27–29]. Furthermore, local guidelines for treating TB and provider adherence to standards of care could vary within the region [9–13]. For instance, in Peru guidelines regarding treatment intermittency in the continuation phase changed over the study period—from 3 times per week to daily dosing [14]. In addition, since patients were not randomized to the different dosing intervals in our study, there may have been unmeasured confounding variables associated with mortality—as can occur in all observational studies [30]. Finally, there were differences in how many patients each center contributed to the study: Haiti (68%), Peru (18%), Brazil (11%), Chile (1.5%), Mexico (1.2%), and Honduras (1.0%) as well as differences in treatment frequency by site with the two largest contributors (Haiti and Brazil) administering treatment 5–7 days/week to all patients, consistent with their local guidelines [11–13].

Other studies have demonstrated that TB treatment given 2–3 days/week in the continuation phase is associated with an increased risk of relapse, treatment failure, and acquired drug resistance compared to treatment 5–7 days/week, most of which were conducted in HIV-negative persons [2, 15–17]. To date, only one randomized controlled clinical trial of TB treatment intermittency among HIV-positive persons has been conducted; it was performed in India and demonstrated superior efficacy and decreased emergence of drug resistance with daily dosing of TB medications throughout treatment [22]. This is also consistent with evidence from observational studies of HIV-negative persons. [15–17]. A retrospective cohort study in India among 292 HIV-TB coinfected patients on atazanavir/ritonavir compared outcomes among persons receiving daily rifabutin or thrice-weekly rifabutin during the TB treatment continuation phase. More individuals in the daily group achieved clinical cure (73.0% vs. 44.1%, P < 0.001), with no significant differences in relapse/recurrence or all-cause mortality between groups. [31] The lack of a difference in all-cause mortality was similar to our findings in the multivariable analysis stratified by study site.

There were several limitations of our study. First, it was an observational cohort study. Although we performed multivariable analyses to adjust for potentially confounding variables, there may have been residual or unmeasured confounding that could affect study findings. There are other ways that we could have adjusted for confounding variables (e.g., inverse probability weighting), but these other approaches are also potentially sensitive to unmeasured confounding. We did not have information regarding TB treatment failure, TB relapse, nor cause of death. In addition, Latin America and the Caribbean include countries that span from low to high-income levels [32, 33], representing a heterogeneous group with varying access to TB diagnostic tests, TB treatment guidelines (even within countries during the period of study, e.g., Peru), and availability of treatment and care resources. However, this reflects the reality of clinical settings in Latin America and throughout the world. Our results are consistent with recent studies [16, 22, 31–33], but additional data are needed to inform the care of persons with HIV-related TB optimally. Prospective, randomized controlled trials of different TB treatment dosing intervals that include different regions in the globe affected by tuberculosis, would help identify the most effective and cost-effective regimens for TB treatment in persons with HIV who concomitantly receive ART.

## Conclusion

Our study is one of the largest studies assessing possible associations between TB treatment intermittency in the continuation phase and mortality among persons with HIV on ART, and the only such study from Latin America and the Caribbean. In multivariable analysis, survival was improved among patients who received 5–7/week dosing. However, after then stratifying by study site, the protective effect was no longer observed. This was perhaps due to heterogeneity in clinical practice at the different study sites or unmeasured confounding. Our study suggests that TB treatment dosing 5–7 days/week during the continuation phase of treatment could reduce mortality in HIV-positive patients, but prospective randomized trials are needed.

## Supplementary Information

**Additional file 1: Table S1.** Clinical and demographic characteristics of those excluded vs. included in the analysis. Table that indicates clinical and demographic characteristics of those excluded vs. included dichotomized by continuation phase treatment. 

## Data Availability

All data generated or analysed during this study are included in this published article.

## Abbreviations

TB: Tuberculosis
ART: Antiretroviral therapy
CCASAnet: Caribbean, Central and South America network for HIV epidemiology
WHO: World Health Organization
CDC: Centers of Disease Control and Prevention
IDSA: Infectious Diseases Society of America
RTC: Randomized controlled trial
IeDEA: International epidemiology Databases to Evaluate AIDS
DCC: Data Coordinating Center
INH: Isoniazid
RIF: Rifampin
RBT: Rifabutin
PZA: Pyrazinamide
EMB: Ethambutol
IQR: Interquartile range
