# Supplementary material for: Tuberculosis treatment intermittency in the continuation phase and mortality in HIV-positive persons receiving antiretroviral therapy
Source: BMC Infect Dis. 2022 Apr 5;22:341. doi: 10.1186/s12879-022-07330-5 (PMC8985331; doi:10.1186/s12879-022-07330-5)
Supplement: Supplementary file 1 — Additional file 1: Table S1. Clinical and demographic characteristics of those excluded vs. included in the analysis. Table that indicates clinical and demographic characteristics of those excluded vs. included dichotomized by continuation phase treatment. [file 12879_2022_7330_MOESM1_ESM.docx]

|  | N | Excluded | Included | Combined | P-value |
| --- | --- | --- | --- | --- | --- |
|  |  | N=2021 | N=3733 | N=5754 |  |
| Sex | 5754 |  |  |  | <0.001 |
| Female |  | 34% (683) | 41% (1531) | 38% (2214) |  |
| Male |  | 66% (1338) | 59% (2202) | 62% (3540) |  |
| Year of TB diagnosis | 5754 | 2014  (2009, 2016) | 2011  (2008, 2013) | 2011  (2008, 2014) | <0.001 |
| Initiation phase TB therapy status | 5754 |  |  |  | <0.001 |
| Received standard initiation TB therapy |  | 76% (1528) | 100% (3733) | 91% (5261) |  |
| No standard initiation TB therapy |  | 24% (493) | 0% (0) | 9% (493) |  |
| Continuation phase TB therapy status  (after excluding initiation phase) | 5754 |  |  |  | <0.001 |
| Received standard continuation TB therapy |  | 24% (493) | 100% (3733) | 73% (4226) |  |
| No standard continuation TB therapy |  | 76% (1528) | 0% (0) | 27% (1528) |  |
| Site | 5754 |  |  |  | <0.001 |
| Brazil |  | 9% (175) | 8% (304) | 8% (479) |  |
| Chile |  | 4% (76) | 2% (67) | 2% (143) |  |
| Haiti |  | 52% (1049) | 67% (2485) | 61% (3534) |  |
| Honduras |  | 3% (56) | 1% (42) | 2% (98) |  |
| Mexico |  | 4% (89) | 2% (75) | 3% (164) |  |
| Peru |  | 29% (576) | 20% (760) | 23% (1336) |  |
| TB location | 5716 |  |  |  | <0.001 |
| Pulmonary only |  | 70% (1397) | 76% (2819) | 74% (4216) |  |
| Any extrapulmonary |  | 30% (598) | 24% (902) | 26% (1500) |  |
| Died | 5754 |  |  |  | <0.001 |
| No |  | 79% (1595) | 86% (3211) | 84% (4806) |  |
| Yes |  | 21% (426) | 14% (522) | 16% (948) |  |

**Table S. Clinical and demographic characteristics of those excluded vs. included in the analysis.**
